# Amiodarone-induced QT prolongation in a newly transplanted heart associated with recurrent ventricular fibrillation

**Published:** 2010-04-01

**Authors:** R ERNST Schwarz, Lawrence S CZER, Sinan A Simsir, Kass M Robert, Alfredo Trento

**Affiliations:** Cedars Sinai Heart Institute, Division of Cardiology, Division of Cardiothoracic Surgery, Comprehensive Transplant Center, Cedars Sinai Medical Center, Los Angeles, and University of California Los Angeles, (UCLA), Los Angeles, USA; Cedars Sinai Heart Institute, Division of Cardiology, Division of Cardiothoracic Surgery, Comprehensive Transplant Center, Cedars Sinai Medical Center, Los Angeles, and University of California Los Angeles, (UCLA), Los Angeles, USA; Cedars Sinai Heart Institute, Division of Cardiology, Division of Cardiothoracic Surgery, Comprehensive Transplant Center, Cedars Sinai Medical Center, Los Angeles, and University of California Los Angeles, (UCLA), Los Angeles, USA; Cedars Sinai Heart Institute, Division of Cardiology, Division of Cardiothoracic Surgery, Comprehensive Transplant Center, Cedars Sinai Medical Center, Los Angeles, and University of California Los Angeles, (UCLA), Los Angeles, USA; Cedars Sinai Heart Institute, Division of Cardiology, Division of Cardiothoracic Surgery, Comprehensive Transplant Center, Cedars Sinai Medical Center, Los Angeles, and University of California Los Angeles, (UCLA), Los Angeles, USA

**Keywords:** ventricular fibrillation, QT prolongation, antiarrhythmic drugs, heart transplantation

## Abstract

Anti-arrhythmic drugs such as amiodarone have the potential to prolong QT intervals, which can result in torsades de point arrhythmia. It is unknown whether amiodarone, given to a recipient prior to cardiac transplantation, can cause arrhythmia in a newly transplanted donor heart. We report on a case of a 71-year-old male patient who had received intravenous and oral amiodarone prior to transplantation, which wasassociated with QT prolongation in the transplanted heart after re-exposure to the drug during subsequent episodes of ventricular fibrillation. An ICD was implanted, which has not been described that soon after cardiac transplantation. Amiodarone, given to a recipient, might cause QT prolongation in a donor heart after transplantation, possibly due to its long half-life and increased bioavailability caused by interaction with immunosuppressive drugs.

## Introduction

Cardiac transplantation represents the ultimate therapeutic solution for end-stage heart failure. Currently, approximately 2 200 heart transplantations are performed in the USA per year.^1^ Survival rates at one, five and 10 years after cardiac transplantation are reported as 87, 77 and 57%, respectively. The average life expectancy after cardiac transplantation is approximately 10 years in adults (9.16 years in a recent study^2^). Quality of life in patients 10 years after heart transplantation has been described as similar to that in the general population.^2^ Arrhythmias early after transplant surgery, in particular paroxysmal atrial fibrillation, have been described in 5% of cases,^3^ even independent of evidence of cellular or humoral rejection.

In a large retrospective analysis, cardiac transplantation – in contrast to CABG surgery in low-risk patients – was considered the strongest independent predictor of freedom from postoperative atrial fibrillation (odds ratio 96; 95% confidence interval: 13–720).^4^ The incidence of atrial fibrillation, atrial flutter and supraventricular tachycardia after cardiac transplantation was reported as 0.33, 2.8 and 1.3%, respectively.^4^ Early postoperative supraventricular arrhythmias are usually of a transient nature and rarely require specific anti-arrhythmic therapy.

In contrast, the potential pro-arrhythmogenic effects of antiarrhythmic drugs are well known.^5^ QT prolongation with subsequent torsade de point arrhythmias, in particular after amiodarone therapy potentially resulting in ventricular fibrillation, has been described repeatedly.^6^-^8^

We report on a patient with end-stage heart failure treated with intravenous amiodarone followed by oral amiodarone prior to cardiac transplantation. With a single intravenous re-exposure to the drug after transplantation for an episode of atrial fibrillation, the patient developed QT prolongation with subsequent ventricular fibrillation, requiring ICD implantation soon after transplant. This has not been reported before.

## Case report

The patient was a 71-year-old male with a long-standing history of cardiovascular disease, coronary artery disease, status post myocardial infarction 14 years ago, who underwent coronary artery bypass grafting seven years earlier with a mammary graft to the left anterior descending artery, a vein graft to the diagonal branch, a vein graft to the marginal branch, and a vein graft to the right coronary artery. Over the past five years, the patient had developed symptomatic heart failure, most likely due to myocardial ischaemia.

Echocardiography demonstrated severe left ventricular dysfunction with an ejection fraction of 16% and severe mitral regurgitation. Coronary angiography showed occlusion of all grafts except the mammary artery graft. Due to severe coronary sclerosis in the native coronary arteries, no interventional or surgical options for revascularisation were considered. After repeated outside hospital admissions for acute decompensated heart failure, the patient was referred in congestive heart failure New York Heart Association class IV, stage D, for advanced heart failure therapy

The patient had a left bundle branch block and a cardioverter/ defibrillator/biventricular pacemaker (CRTD) device was implanted for cardiac resynchronisation therapy and sudden death prophylaxis. Due to persistent symptoms of heart failure despite optimised medical and electrical therapy, the patient was evaluated for cardiac transplantation. A right heart catheterisation revealed a PA pressure of 68/38 mmHg with a mean of 49 mmHg and pulmonary capillary wedge pressures of 41/64 mmHg with a mean of 49 mmHg, and a cardiac output of 1.63 l/min with a cardiac index of 0.89 l/min/m^2^.

Continuous intravenous (IV) inotropic therapy using dobutamine was initiated at 5 mcg/kg/min in combination with IV diuretics. The cardiac index improved to 1.8 l/min/m^2^, and with persistent symptoms of heart failure after completing the evaluation process, the patient was listed for cardiac transplantation as a status IB.

Even before dobutamine therapy, the patient had recurrent episodes of ventricular tachycardia, supraventricular tachycardia and intermittent atrial fribrillation. Amiodarone therapy was initiated with an initial bolus of 150 mg IV, followed by continuous infusion of 1 mg/min for six hours, followed by 0.5 mg/min for 18 hours as a continuous infusion, and then changed to oral amiodarone 200 mg given twice daily 12 hours apart.

**Fig. 1.**
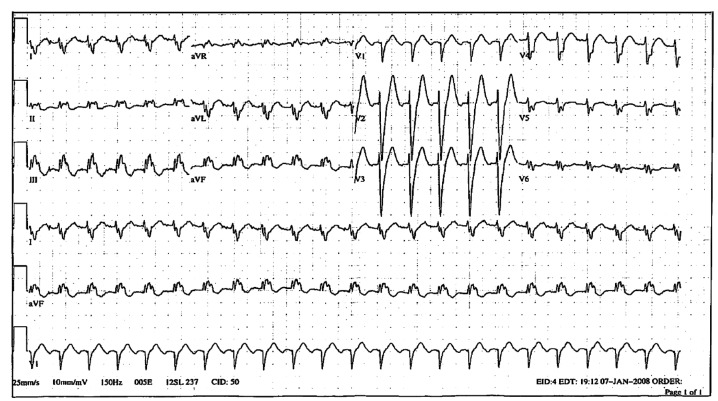
ECG 1 of the patient’s heart directly prior to surgery (January 4), after treatment with amiodarone, showing sinus tachycardia with a rate of 134/min, right axis deviation and left bundle branch block. QT duration is 346 msec.

The patient’s rhythm stabilised (Fig. 1) and after 10 days, orthotopic cardiac transplantation was performed successfully. The donor heart was from a 36-year-old male without any prior cardiovascular history or prior anti-arrhythmic therapy. The donor’s ECG was without any abnormalities.

**Fig. 2.**
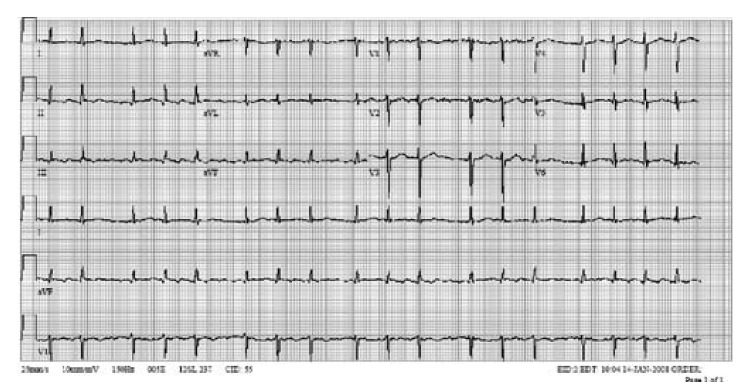
ECG 2 of the same patient seven days after transplantation (January 11) of the donor heart, showing paroxysmal atrial fibrillation and heart rate approximately 105/min. QT duration is 344 msec.

**Fig. 3.**
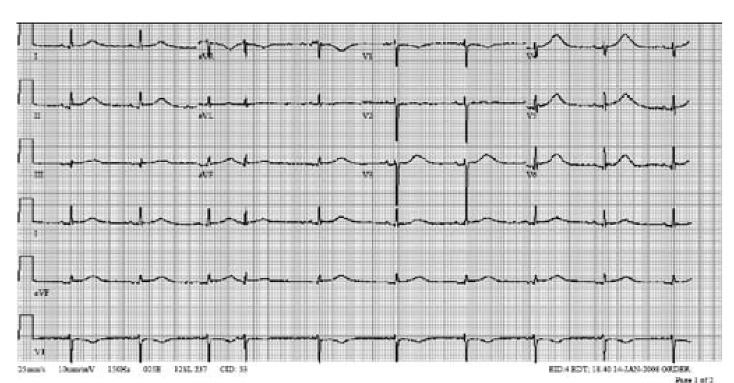
ECG 3 of the recipient after cardiac transplantation (January 13) with sinus rhythm, heart rate 60/min and prolonged QT interval of 550 msec. Shortly thereafter, the patient developed ventricular fibrillation.

On postoperative day seven, the recipient developed paroxysmal atrial fibrillation (Fig. 2). A single amiodarone bolus of 150 mg was given, followed by an infusion of 0.5 mg/min and the patient converted to sinus rhythm. Subsequently, the patient demonstrated prolonged QT interval on ECG (550 msec, QTc 544 msec, Fig. 3) and developed torsade de pointes, which degenerated into ventricular fibrillation, with successful external defibrillation.

Amiodarone was discontinued, electrolytes were in the normal range but IV magnesium was started for a borderline low magnesium level of 1.5 mg/dl. Despite shorter but still prolonged QT intervals the following day (454 ms, QTc 517 msec) with normal electrolytes, the patient had another episode of ventricular fibrillation, which required external defibrillation. Subsequent ECGs demonstrated normalised QT intervals.

Cardiac function, assessed by transthoracic two-dimensional echocardiography was normal, and a coronary angiogram demonstrated normal coronary arteries without angiographic evidence of any obstructive disease, coronary spasm or embolus. Right ventricular endomyocardial biopsies did not reveal any signs of acute cellular or humoral rejection.

We hypothesise that amiodarone given to the recipient for a prolonged time prior to transplantation, followed by re-exposure to intravenous amiodarone with increased bioavailability due to concomitant tacrolimus therapy after transplantation might have caused prolonged QT interval in the donor heart with subsequent ventricular arrhythmia.

After considering a watch-and-wait approach versus a temporary wearable defibrillator vest (which was rejected by the patient) it was decided to proceed with implantation of a single-chamber ICD as primary prevention of sudden death 14 days after cardiac transplantation. The patient was discharged in a stable condition and so far, has had no further arrhythmic events.

## Discussion

We report recurrent ventricular fibrillation in a patient soon after cardiac transplantation without evidence of electrolyte abnormalities, myocardial ischaemia, humoral or cellular rejection or graft dysfunction. ECG revealed a prolonged QT interval, which occurred after a brief re-exposure to intravenous amiodarone after the recipient had had prolonged IV and oral amiodarone in the days prior to transplantation.

Even though not proven without doubt, it is likely that previous amiodarone therapy induced QT prolongation in the transplanted donor heart due to its long half-life, even after the drug was stopped.^9^,^10^ The brief intravenous re-exposure might have boosted amiodarone levels, however, unlike oral amiodarone, IV amiodarone alone is rarely associated with significant QT prolongation and even more rarely with torsade de pointes.

Other potential reasons for the QT prolongation cannot be ruled out completely. A cross check of drugs known to prolong QT intervals, such as albuterol, alfuzosin, amantadine, amitriptyline, amphetamines, arsenic, astemizole, atazanavir, atomoxetine, azythromycin, chloroquine, clomipramine, dolasetron, metaproterenol, moxifloxacin, phentermine, and phenylpropanolamine revealed that none of those had been reported to be taken by the recipient or the donor in the past.

There was no significant bradycardia and no relevant electrolyteabnormalities that could have caused either QT prolongation or ventricular arrhythmia. No evidence for myocardial ischaemia, graft dysfunction or rejection was evident. The donor had no known history of long QT syndrome or any cardiovascular disorder and had died of non-cardiac reasons. Therefore, we believe that the QT-prolonging and pro-arrhythmic potential of extended amiodarone therapy prior to transplant caused the events in the case described here.

In general, the occurrence of QT prolongation with subsequent ventricular fibrillation in the absence of ischaemia or graft rejection is extremely rare early after cardiac transplantation, and in fact, has not been reported so far, to the best of our knowledge. Also, we did not find any reports in the literature of either ventricular fibrillation or ICD implantation in the early postoperative period after heart transplantation or on potential anti-arrhythmia-induced QT prolongation.

Serum levels of amiodarone, even though not measured, could have been increased by a potential drug interaction with tacrolimus,^11^ which was given as part of the immunosuppressive regime after transplantation. Similarly, this has been described with cyclosporine,^12^ and both drugs can contribute to higher bioavailability of amiodarone.

MacDonald et al. demonstrated that patients who received amiodarone before transplantation had significantly lower heart rates at one and four weeks after transplantation and required prolonged atrial pacing (7.3 ± 1.4 versus 3.4 ± 0.8 days, p < 0.02). In a relatively small study of 19 patients on amiodarone compared to 31 controls, no effect of prior amiodarone therapy on early allograft function or on clinical outcome was detected.^13^

Even though some authors in the past have recommended continuing amiodarone after cardiac transplantation because of serious side effects after discontinuation of the drug,^14^ this is not recommended anymore. Moreover, there is also controversy about the use of beta-adrenergic blockers (early) after transplantation, 15 but there are no data from controlled large-scale studies.

Since we believed that the arrhythmia was most likely caused by amiodarone, we recommended waiting until the drug was completely cleared, as demonstrated by continuous monitoring. Also, a wearable defibrillator vest was discussed, but neither was accepted by the patient and therefore, a single-chamber ICD was implanted for the prevention of sudden death. This has not been reported that early after transplantation before.

In contrast, ventricular arrhythmias and the need for antiarrhythmic therapy, including ICD implantation has been described late after cardiac transplantation. Arrhythmic events in the post-transplant population are most often associated with graft dysfunction secondary to chronic allograft vasculopathy and subsequent myocardial ischaemia, with an increased risk of sudden death years after transplant.^16^ The usefulness of ICD implantation has been described in 10 out of 493 heart transplant recipients,^17^ which is not as common as ICD implantation in heart failure patients awaiting cardiac transplantation.^18^-^20^

## Conclusion

The potential arrhythmogenic effects of anti-arrhythmic drugs such as amiodarone should be considered, even after cessation of the drug, and even in the transplanted donor heart. Because of the potential drug interactions with immunosuppressive agents such as tacrolimus, the QT interval in the transplanted heart should be monitored closely in patients who had received amiodarone prior to transplantation.

## References

[1] Fraund S, Pethig K, Franke U, Wahlers T, Harringer W, Cremer J, Fieguth HG, Oppelt P, Haverich A (1999). Ten year survival after heart transplantation: palliative procedure or successful long term treatment?. Heart.

[2] Politi P, Piccinelli M, Poli PF, Klersy C, Campana C, Goggi C, Viganò M, Barale F (2004). Ten years of ‘extended’ life: quality of life among heart transplantation survivors.. Ten years of ‘extended’ life: quality of life among heart transplantation survivors..

[3] Cohn WE, Gegoric ID, Radovancevic B, Wolf RK, Frazier OH (2008). Atrial fibrillation after cardiac transplantation: experience in 498 consecutive cases.. Ann Thorac Surg.

[4] Khan M, Kalahasti V, Rajagopal V, Khaykin Y, Wazni O, Almahameed S (2006). Incidence of atrial fibrillation in heart transplant patients: longterm follow-up.. J Cardiovasc Electrophysiol.

[5] Lazzara R (1993). Antiarrhythmic drugs and torsade de pointes.. Eur Heart J.

[6] McComb JM, Logan KR, Khan MM, Geddes JS, Adegey AA (1980). Amiodaroneinduced ventricular fibrillation.. Eur J Cardiol.

[7] Hii JT, Wyse DG, Gillis AM, Duff HJ, Solylo MA, Mitchell LB (1992). Precordial QT interval dispersion as a marker of torsade de pointes. Disparate effects of class Ia antiarrhythmic drugs and amiodarone.. Circulation.

[8] Schrickel J, Bielik H, Yang A, Schwab JO, Shlevkov N, Schimpf R (2003). Amiodarone-associated ‘torsade de pointes’. Relevance of concomitant cardiovascular medication in a patient with atrial fibrillation and structural heart disease.. Z Kardiol.

[9] Zipes DP, Prystowsky EN, Heger JJ (1984). Amiodarone: electrophysiologic actions, pharmacokinetics and clinical effects.. J Am Coll Cardiol.

[10] (2006). Adverse effects of amiodarone: even after the end of treatment.. Prescrire Int.

[11] Nalli N, Stewart-Teixeira L, Dipchand AI (2006). Amiodarone-sirolimus/tacrolimus interaction in a pediatric heart transplant patient.. Pediatr Transplant.

[12] Chitwood KK, Abdul-Haqq AJ, Heim-Duthoy KL (1993). Cyclosporineamiodarone interaction.. Ann Pharmacother.

[13] Macdonald P, Hackworthy R, Keogh A, Sivathasan C, Chang V, Spratt P (1991). The effect of chronic amiodarone therapy before transplantation on early cardiac allograft function.. J Herat Lung Transplant.

[14] Preuner JG, Lehle K, Keyser A, Merk J, Rupprecht L, Goebels R (1998). Development of severe adverse effects after discontinuing amiodarone therapy in human heart transplant recipients.. Transplant Proc.

[15] Verani MS, Nishimura S, Mahmarian JJ, Hays JT, Young JB (1994). Cardiac function after orthotopic heart transplantation: response to postural changes, exercise, and beta-adrenergic blockade.. J Heart Lung Transplant.

[16] Weiss MJ, Madsen JC, Rosengard BR, Allan JS (2008). Mechanisms of chronic rejection in cardiothoracic transplantation.. Front Biosci.

[17] Ptaszek LM, Wang PJ, Hunt SA, Valantine H, Perlroth M, Al-Ahmad A (2005). Use of the implantable cardioverter-defibrillator in long-term survivors of orthotopic heart transplantation.. Heart Rhythm.

[18] Lorga-Filho A, Geelen P, Vanderheyden M, Malacky T, Primo J, Goethals M (1998). Early benefit of implantable cardioverter defibrillator therapy in patients waiting for cardiac transplantation.. Pacing Clin Electrophysiol.

[19] Grimm M, Grimm G, Zuckermann A, Wieselthaler G, Feuerstein M, Daneschvar H (1995). ICD therapy in survivors of sudden cardiac death awaiting heart transplantation.. Ann Thorac Surg.

[20] Podczeck A, Hief C, Jakl G, Frohner K, Nürnberg M, Kaltenbrunner W (1995). [Efficacy of the implantable cardioverter-defibrillator in patients on the waiting list for heart transplantation.]. Wien Klin Wochenschr.

